# Supplementary figures and images for: Binding of transmissible gastroenteritis virus and porcine respiratory coronavirus to human and porcine aminopeptidase N receptors as an indicator of cross-species transmission
Source: PLoS One. 2025 May 27;20(5):e0325023. doi: 10.1371/journal.pone.0325023 (PMC12111490; doi:10.1371/journal.pone.0325023)

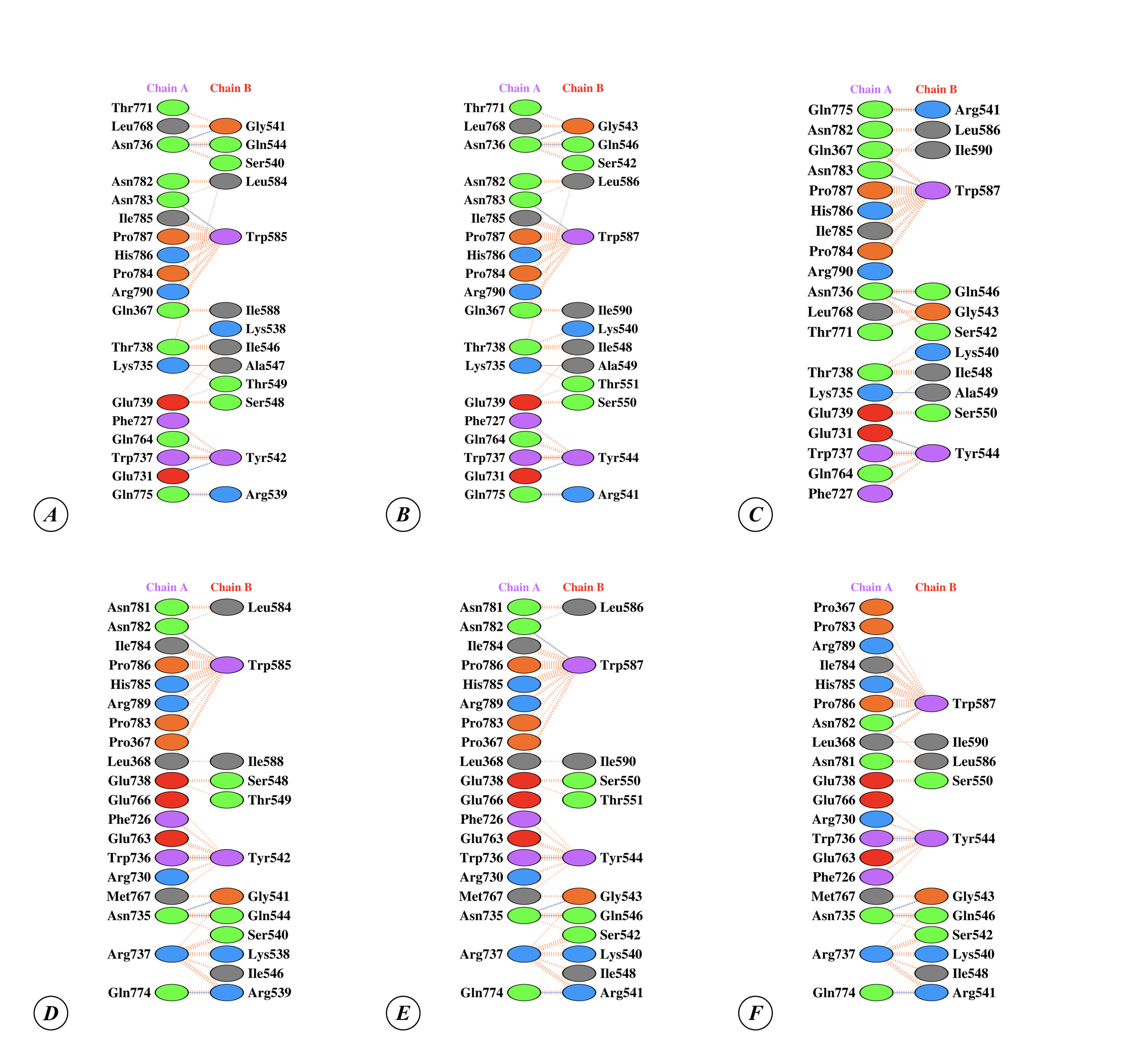

Supplement: S5 Fig — (A–C) Interactions between pig APN and RBDs of the Purdue strain (A), 133 strain (B), and 133 strain with additional mutations (C). (D–F) Interactions between human APN and RBDs of the Purdue strain (D), 133 strain (E), and 133 strain with additional mutations (F). Chain A corresponds to APN receptors, and chain B to RBDs. H-bonds are represented as blue lines, while non-bonded contacts are depicted as orange striped lines, with stripes’ width proportional to the number of atomic contacts. Positive residues are colored blue, negative residues red, neutral residues green, aliphatic residues gray, aromatic residues purple, and proline and glycine residues orange. (TIF) [file pone.0325023.s005.tif]
