# Supplementary material for: Binding of transmissible gastroenteritis virus and porcine respiratory coronavirus to human and porcine aminopeptidase N receptors as an indicator of cross-species transmission
Source: PLoS One. 2025 May 27;20(5):e0325023. doi: 10.1371/journal.pone.0325023 (PMC12111490; doi:10.1371/journal.pone.0325023)
Supplement: Attachment — Submitted filename: Response to Reviewers PONE-D-25-05046.pdf [file pone.0325023.s009.pdf]

Dear Editor,

We appreciate the time and effort the Editorial team have dedicated to reviewing our manuscript, "Binding of transmissible gastroenteritis virus and porcine respiratory coronavirus to human and porcine aminopeptidase N receptors as an indicator of cross-species transmission" (PONE-D-25-05046), submitted to PLOS ONE. We respect your decision; however, we would like to formally appeal the rejection and kindly request a reconsideration of our manuscript. We believe that our study aligns with the journal's scope and publication criteria, and we would like to clarify specific aspects that may have contributed to the initial decision.

The primary reason for rejection, as stated in the decision letter, is that our study relies on *in silico* methods and does not include biochemical or virological experiments. However, we would like to highlight that PLOS ONE has previously published purely *in silico* studies in the fields of virology and computational biology. Our work follows a rigorous computational methodology, including phylogenetic analysis, molecular modeling, binding free energy calculations, and molecular dynamics simulations, that are established techniques in computational biology. These approaches have been widely used in reputable published literature to study host-virus interactions and assess infectious potential of viruses.

We believe that our study meets PLOS ONE's criteria for publication, as it provides novel insights into the potential for interspecies transmission of coronaviruses and adheres to established methodological standards in computational biology. Before submitting our manuscript, we carefully reviewed PLOS ONE's policies, publication criteria, and submission guidelines, as well as previously published studies in the journal. Based on this review, we concluded that our study falls within the journal's scope and that our manuscript complies with all formatting and data reporting requirements.

We acknowledge that there may have been some misunderstandings regarding the study's objectives and the implications of our computational findings. To address this, we have revised some sections of our manuscript to more clearly emphasize that our study focuses on assessing coronavirus-receptor interactions using computational methods and that any conclusions drawn about potential interactions remain within

the scope of our results. We have noted the corresponding changes in the "Track Changes" version of our manuscript. We fully acknowledge that experimental validation would further strengthen the findings, and we have carefully framed our conclusions within the scope of *in silico* predictions, avoiding overstatement of biological implications. We believe that this study provides a valuable framework that can guide future laboratory research in the field.

We kindly request a reevaluation of our manuscript, either by reconsidering its suitability for peer review or by providing further clarification on the journal's policy regarding *in silico* research. If additional revisions or clarifications would make the study more appropriate for consideration, we would be happy to make those adjustments.

We sincerely appreciate your time and consideration and look forward to your response.

Best regards,
Mykyta Peka
on behalf of co-authors
