# Supplementary material for: Binding of transmissible gastroenteritis virus and porcine respiratory coronavirus to human and porcine aminopeptidase N receptors as an indicator of cross-species transmission
Source: PLoS One. 2025 May 27;20(5):e0325023. doi: 10.1371/journal.pone.0325023 (PMC12111490; doi:10.1371/journal.pone.0325023)
Supplement: Attachment — Submitted filename: Response to Reviewers_v3.pdf [file pone.0325023.s010.pdf]

Response to Editor:

The authors thank the PLOS ONE editorial board for reviewing the submitted manuscript. The authors have carefully considered the comments and suggestions provided by the editors and reviewers and have undertaken the necessary revisions to ensure that the manuscript meets the high quality standards and the PLOS ONE's publication criteria.

1. The manuscript has been checked to ensure full compliance with PLOS ONE's style requirements.

2. We would like to state that the funder had no role in study design, data collection and analysis, decision to publish, or preparation of the manuscript. As there was no option to do this in Editorial Manager while revising the manuscript, we ask to add an additional sentence to our financial disclosure statement and present it the following form:

"This study was funded by the National Academy of Agrarian Sciences of Ukraine (grant registration number: 0124U002088). The funder had no role in study design, data collection and analysis, decision to publish, or preparation of the manuscript."

3. A point-by-point response has been provided to address each of the reviewers' comments, and the necessary revisions of the manuscript have been made.

4. **The authors consent to the publication of the peer review history** of this manuscript in accordance with PLOS ONE's publication policies (https://journals.plos.org/plosone/s/editorial-and-peer-review-process#loc-peer-review-history).

The authors remain fully committed to collaborating with the PLOS ONE editorial board to ensure that the manuscript meets the necessary quality standards.

Response to Reviewer 1:

The authors thank the Reviewer for evaluation of this manuscript, suggestions, and comments. We have addressed the following points raised in the review to enhance the clarity and overall quality of the manuscript:

Reviewer #1: here is my comments on "Binding of transmissible gastroenteritis virus and porcine .." Manu.
the manuscript needs improvements in deiffernt aspects; as follows

(1) While the authors have effectively used Grantham's Distance (Table 1) to assess the conservatism or radicality of amino acid substitutions, incorporating multiple sequence alignment (MSA) could further enhance the analysis by providing insights into the evolutionary conservation of these residues across different species. This would help in determining whether the observed substitutions occur in highly conserved regions, which could indicate functional significance.

Authors' response. The authors fully agree with the Reviewer's observation that multiple alignment can enhance the depth of the analysis. In response, an additional multiple alignment was performed, incorporating spike protein sequences from other *Coronaviridae* family members that are relevant to the study of human and animal infections. These include canine coronavirus (CCoV) and HCoV-229E, both alphacoronaviruses that utilize the APN receptor, similar to TGEV and PRCV, as well as SARS-CoV, SARS-CoV-2, and MERS-CoV, which are betacoronaviruses responsible for human epidemics and utilize different receptors. Additionally, porcine deltacoronavirus (PDCoV), an emerging porcine enteropathogenic virus that, despite being distinct from TGEV, also utilizes APN, was included. The results of this alignment are presented in Fig 2.

Conducting this multiple alignment not only provided additional valuable insights into the interspecies conservation of the spike protein at the RBD but also allowed for the optimization of result presentation. Fig 2 now more clearly illustrates the positions of amino acid substitutions both between TGEV and PRCV strains and at the interspecies level. Furthermore, by incorporating a color panel based on Grantham's distance categorization (conservative, moderately conservative, moderately radical, and radical substitutions), the conservation of these substitutions is effectively highlighted.

Given these enhancements, the authors concluded that Fig 2 presents the findings more comprehensively than the previously included table and therefore deserves placement in the main part of the manuscript. The table containing Grantham's distance estimates for substitutions at specific positions, which was originally presented in this context, has been converted into an editable tabular format and relocated to the Supporting data files (S2 Table), ensuring it remains accessible for interested readers.

The authors express their sincere gratitude to the Reviewer for this insightful suggestion. Implementing multiple alignment not only led to additional significant findings and a deeper discussion but also improved the clarity and structure of results presentation.

(2) The authors have utilised the Hawdock server for their binding free energy calculations. However, an alternative approach such as gmx_MMPBSA, which integrates molecular dynamics simulations with MM/PBSA calculations in GROMACS, could provide a more dynamic and detailed estimation of ΔG. Could the authors clarify why they opted for Hawdock instead of employing gmx_MMPBSA for free energy calculations?"

Authors' response. The authors would like to highlight that both the Hawkdock server and the gmx_MMPBSA approach were used in this study for binding free energy calculations. The Hawkdock server was employed for the initial analysis and comparison of different TGEV and PRCV strains, as well as for screening theoretical mutations that could enhance the affinity of the RBD to pig and human APN-receptors. The binding free energy calculations performed using the Hawkdock server are based on the MM/GBSA approach, which is quite accurate. Additionally, the Hawkdock server allows for a balance between computational efficiency and accuracy, which was important for the comparative analysis of different strains and the screening of the set of potential mutations.

Furthermore, the gmx_MMPBSA approach was applied to assess the binding free energy of RBD-APN complexes selected for molecular dynamics simulations. As correctly noted by the Reviewer, this method enabled a more detailed estimation of ΔG in a dynamic context, accounting for potential changes in ΔG associated with conformational changes during molecular dynamics simulations.

The authors have made every effort to clearly specify in the Materials and Methods section the contexts in which each approach (Hawkdock server or gmx_MMPBSA) was used for ΔG calculations.

(3) The manuscript would benefit from additional graphical representations of molecular interactions. More detailed visualizations of key interactions, such as hydrogen bonds, hydrophobic contacts, and salt bridges, could enhance the clarity of the findings.

Authors' response. In accordance with the recommendations, the authors have expanded the graphical presentation. In addition to the general view of the RBD-APN complex (Fig 3A), the manuscript now includes images illustrating the interface interaction of the virus's RBD with pAPN (Fig 3B) and hAPN (Fig 3C). Furthermore, the authors would like to notice that a detailed schematic representation of the various interface contacts in the RBD-APN complexes is available in supplementary figure (S5 Figure).

(4) Regarding the calculated ΔΔG values for the mutations, could the authors clarify whether these calculations were performed using the PDB structures before or after molecular dynamics (MD) simulations? Using post-MD structures could provide a more realistic representation of the mutated protein's stability and interactions.

Authors' response. In our study, ΔΔG calculations for single amino acid mutations were performed using reference structures based on the conformation and

orientation of the RBD and APN in a previously defined crystallographic RBD-APN complex (PDB: 4F5C). This approach ensured structurally reliable starting points for mutation screening and maintained consistency in the calculations. ΔΔG prediction tools are designed to operate with static structures rather than molecular dynamics (MD)-derived ensembles, making this a common approach in stability prediction studies.

Following MD simulations of selected complexes, instead of recalculating ΔΔG, we computed the ΔG values for these complexes throughout the MD simulations. This approach provides comparative insights into the stability of complexes over time, effectively capturing the same information as post-MD ΔΔG calculations. Since ΔΔG is defined as the difference between two ΔG values ($\Delta\Delta G = \Delta G_2 - \Delta G_1$), tracking ΔG values across MD also allowed us to assess the relative stability of complexes dynamically.

(5) The authors have calculated ΔG in the gas phase. Could they explain why this approach was used, considering that molecular interactions typically occur in aqueous environments?

Authors' response. The authors agree with the Reviewer that molecular interactions occur in aqueous environments and would like to emphasize that solvation effects were accounted for in binding free energy calculations. Both the Hawkdock server and gmx_MMPBSA tool incorporate solvation effects in their methodologies. Specifically, the Hawkdock server accounts for polar and non-polar solvation contributions when calculating ΔG.

For ΔG values derived using gmx_MMPBSA tool based on molecular dynamics simulations, the authors have reported several energy components in the figure titled "Analysis of virus binding to APN through molecular dynamics." This includes molecular mechanics energy in the gas phase (Figure 5G-H), solvation energy contributions (Figure 5I-J), and total binding free energy (Figure 5E-F). Additionally, in the current version of the manuscript, Table 8 has been added to provide a detailed breakdown of each energy component, including molecular mechanics (bonded interactions, van der Waals forces, electrostatic and non-bonded interactions), as well as polar and non-polar solvation energy contributions to the total binding free energy.

Thus, the binding free energy calculations account for both molecular mechanics energy in the gas phase and solvation effects.

The authors sincerely thank the Reviewer once again for all valuable recommendations, which have significantly improved the quality of this manuscript. We remain fully committed to making any refinements if necessary to ensure that the manuscript meets the high quality standards.

Response to Reviewer 2:

Reviewer #2: This manuscript shows novel aspects of TGEV and PRCV at the possibility of zoonotic pathogens.

The following points should be considered for the clearness of this manuscript.

line 198 to 199 : on the on the HawkDock... --> on the HawkDock...

Authors' response. The authors are grateful to the Reviewer for evaluation of the manuscript. The authors made adjustments and removed duplication in the line:

...on the on the HawkDock... --> on the HawkDock...

We would like to emphasize that, during the revision process, the manuscript was thoroughly proofread to ensure that it is well-written, with particular attention to language accuracy and clarity of presentation. We remain fully committed to making any refinements if necessary to ensure that the manuscript meets the high quality standards.
