# Supplementary material for: Binding of transmissible gastroenteritis virus and porcine respiratory coronavirus to human and porcine aminopeptidase N receptors as an indicator of cross-species transmission
Source: PLoS One. 2025 May 27;20(5):e0325023. doi: 10.1371/journal.pone.0325023 (PMC12111490; doi:10.1371/journal.pone.0325023)
Supplement: Attachment — Submitted filename: Resopnse to reviewers_v4.pdf [file pone.0325023.s011.pdf]

Response to Editor:

The authors thank the PLOS ONE Editorial Board for their time and effort in reviewing the submitted manuscript. We appreciate the valuable comments provided by the Editor and Reviewers, which have contributed significantly to enhancing the quality of the manuscript.

In accordance with the Editor's instructions, the reference list was carefully reviewed to ensure its accuracy. We also confirm that it does not include citations of any retracted sources.

In response to the Reviewer's suggestion regarding figure formatting for publication, we would like to note that all figures have been processed using the Preflight Analysis and Conversion Engine (PACE) digital diagnostic tool, as recommended. This step ensures that the figures fully comply with PLOS formatting requirements.

The authors reaffirm their commitment to continued collaboration with the Editorial Board and Reviewers to maintain the high quality of the manuscript and ensure its full adherence to PLOS ONE's publication standards.

Response to Reviewer 1:

The authors thank the Reviewer for the time dedicated to re-evaluating the revised manuscript. In response to the Reviewer's comments, the images included in the manuscript were carefully reviewed to ensure that all titles and labels are free of grammatical errors, and that the images fully comply with the PLOS ONE submission guidelines, including those related to format, resolution, dimensions, etc.

Following the Reviewer's recommendation to represent the interactions by the contribution of forces, the authors have added Figure 6 (numbered accordingly in the current version of the manuscript). This figure illustrates the dynamics of multiple interaction components, including van der Waals interaction energy, electrostatic interaction energy, and both polar and non-polar solvation energy components. These additions enhance the informational value and presentation of the results.

The authors are grateful for the Reviewer's constructive suggestions, which have helped improve the overall quality of the manuscript. We remain committed to continued collaboration with the Editorial Board and Reviewer throughout the editorial process.
